# Delving into the pinnacle: an in-depth analysis of the top 100 most cited articles on cardio-oncology

**DOI:** 10.1186/s41065-025-00497-2

**Published:** 2025-07-16

**Authors:** Ping Lai, Shuquan Xu, Zi-xuan Cao, Zi-you Liu, Ke-jun Tian, Wen-ting Zhong, Xia Liu, Yi-ming Zhong, Xiao-ping Wang, Jin-hua Xue

**Affiliations:** 1 Department of Cardiology, The First Affiliated Hospital of Gannan Medical University, Gannan Medical University, Ganzhou, Jiangxi Province 341000 China; 2 Key Laboratory of Prevention and Treatment of Cardiovascular and Cerebrovascular Diseases, Ministry of Education, Gannan Medical University, Ganzhou, 341000 China; 3 The First School of Clinical Medicine, Gannan Medical University, Ganzhou, Jiangxi Province 341000 China; 4 Heze Central Blood Station, Heze, Shandong Province 274000 China; 5 Heart Medical Centre, First Affiliated Hospital of Gannan Medical University, Ganzhou, Jiangxi Province 341000 China; 6 Department of Oncology, The First Affiliated Hospital of Gannan Medical University, Gannan Medical University, Ganzhou, Jiangxi Province 341000 China; 7 Department of Physiology, School of Basic Medical Sciences, Gannan Medical University, Ganzhou, 341000 China; 8 Department of Physiology, School of Basic Medical Sciences, Key Laboratory of Prevention and Treatment of Cardiovascular and Cerebrovascular Diseases, Ministry of Education, Gannan Medical University, Ganzhou, 341000 China; 9 The First Affiliated Hospital of Gannan Medical University, Gannan Medical University, No.23 of Qingnian Road, Ganzhou, Jiangxi Province 341000 China

**Keywords:** Most cited, Cardio-oncology, Management, VOSviewer, Citespace

## Abstract

The intersection of myocardial diseases and cancer therapy-induced cardiovascular toxicity has garnered significant attention in recent years. As the field of cardio-oncology bridges cardiology and oncology, research is expanding rapidly. This study analyzes the top 100 most cited papers (T100) in cardio-oncology, providing valuable insights into current trends and key research areas. Using Microsoft Excel 2009, we conducted a comprehensive temporal analysis of publications and citations. To visualize complex networks involving co-citations, countries, and author keywords, we utilized VOSviewer software. Additionally, Citespace was employed to identify clusters of keywords experiencing notable citation surges. The T100 selection includes 81 clinical research papers and 19 foundational studies. Key journals such as the European Heart Journal and the Journal of the American College of Cardiology stand out, collectively accumulating over 700 citations (9 papers from European Heart Journal and 7 from Journal of the American College of Cardiology). Notably, the United States leads the field with 41 publications and 2,868 citations, excluding self-citations. Prominent keywords emerging in this area include “cardiovascular diseases” (strength 2.48, 2019–2022), “management” (average publication year: September 2019, occurrence: 11 times), and “immune checkpoint inhibitors” (average publication year: 2020, occurrence: 5 times). While the T100 predominantly comprises clinical research, there is a discernible gap in foundational studies exploring the mechanisms of myocardial diseases induced by cancer therapy-related cardiovascular toxicity. The USA continues to dominate this field. Research in cardio-oncology focuses on cardiovascular diseases, especially heart failure and atrial fibrillation, with an emphasis on comprehensive management strategies and the potential of immune checkpoint inhibitors. This study offers valuable insights that could enhance our understanding and management of cardio-oncological complications.

## Introduction

Cancer and cardiovascular disease are the leading causes of mortality worldwide [1, 2]. In response to these major health challenges, the past decade has seen the rise of cardio-oncology as a distinct discipline dedicated to addressing cancer therapy-related cardiovascular toxicity (CTR-CVT). This evolving field encompasses a broad spectrum of cardiovascular complications arising from various anti-cancer treatments, including chemotherapy, targeted therapies, immunotherapy, and radiation therapy. These complications include cardiomyopathy, heart failure, myocarditis, vascular toxicities, hypertension, arrhythmias, as well as pericardial and valvular heart diseases [3]. Moreover, the mortality associated with cardiovascular diseases (CVDs) experienced a dramatic 20-fold increase between 2000 and 2016 [4]. Importantly, cancer patients with coexisting CVDs demonstrate significantly higher overall mortality compared to those without such comorbidities [5].

Cardio-oncology has attracted significant global attention, evidenced by a rapid increase in publications over the past five years. Prior to 2017, the annual publication count was fewer than 100, but since 2020, it has exceeded 250 per year [6]. The majority of these studies focus on identifying and mitigating risk factors associated with CTR-CVT. While some risk factors overlap between CVDs and CTR-CVT [7], others are specific to particular groups of cancer patients and cannot be generalized across different malignancies [8]. For example, an early rise in cardiac troponin I levels has been associated with an increased risk of cardiotoxicity in patients undergoing doxorubicin and trastuzumab therapy [9]. Similarly, elevated cardiac troponin T levels have been linked to major adverse cardiac events following immune checkpoint inhibitor (ICI) therapy [10]. Additionally, the use of natriuretic peptides and echocardiography has facilitated the early detection of diastolic dysfunction in otherwise asymptomatic, low-risk cancer patients receiving standard-dose chemotherapy [11]. These findings were also referenced in the most recent European Society of Cardiology (ESC) guidelines [3].

Given the complexity of the molecular mechanisms and pathophysiology involved, along with the diverse range of therapies used in cancer treatment and their associated complications, the absence of targeted treatments for CTR-CVT presents a significant challenge. Consequently, a coordinated, multidisciplinary approach and specialized cardio-oncology services are essential for cancer patients experiencing acute or evolving CTR-CVT during or after cancer treatment [12]. It is important to note that the management of coronary syndromes in cancer patients closely mirrors that of patients without cancer [13]. However, in cancer patients, the treatment of acute coronary syndromes requires careful evaluation of the risks of thrombosis and bleeding [14]. Although the CHA2DS2-VASc score is used to assess atrial fibrillation in cancer patients, its validation remains a focus of ongoing research [15]. In conclusion, the lack of specific treatment options for cancer patients with distinct cardiovascular diseases underscores the urgent need for further research to develop more targeted and personalized therapeutic strategies [16].

Effective prevention is crucial in reducing cardiovascular complications associated with cancer treatment. Prevention strategies include assessing cardiovascular risk factors, conducting baseline cardiac evaluations, promoting lifestyle modifications, and carefully administering cardioprotective medications [17]. Angiotensin-converting enzyme inhibitors and beta-blockers are particularly valuable therapies, as they help manage chemotherapy-induced hypertension, provide cardioprotection by reducing oxidative stress and heart remodeling, and aid in the management of heart failure and arrhythmias [18].

A research paper, by its nature, provides a comprehensive analysis, evaluation, or interpretation of a specific subject. Bibliometric analysis is a quantitative method used to analyze and visualize patterns within a body of literature, focusing on the relationships, trends, and impact of publications in a given field. Unlike traditional literature reviews, which are typically narrative and qualitative, bibliometric analysis uses data-driven techniques to uncover the structure and evolution of research topics over time. Studying highly cited papers offers valuable insights into the evolving dynamics and critical developments within a given field. These papers often highlight pivotal topics, either by representing foundational studies that shape clinical practice or by identifying emerging areas for future research. Bibliometric analysis, a quantitative approach to exploring relevant literature, reveals the bibliometric characteristics of publications within a defined scope [19]. Modern applications of bibliometric analysis, including tools like VOSviewer and Citespace, are widely used to uncover current research trends, hotspots, and potential future directions across various domains, such as internal medicine [20], and stem cell research [21]. Despite their significance, a bibliometric analysis of top-cited papers in cardio-oncology has yet to be conducted, leaving a gap in understanding key research areas and informing clinical practice within this domain.

In this study, we use bibliometric analysis to explore the landscape of research, identify key focal points, and highlight potential avenues of investigation within the field of cardio-oncology by examining the top 100 most cited papers (T100). This analysis serves as a foundation for future research endeavors.

## Materials and methods

### Data extraction

**Fig. 1:**
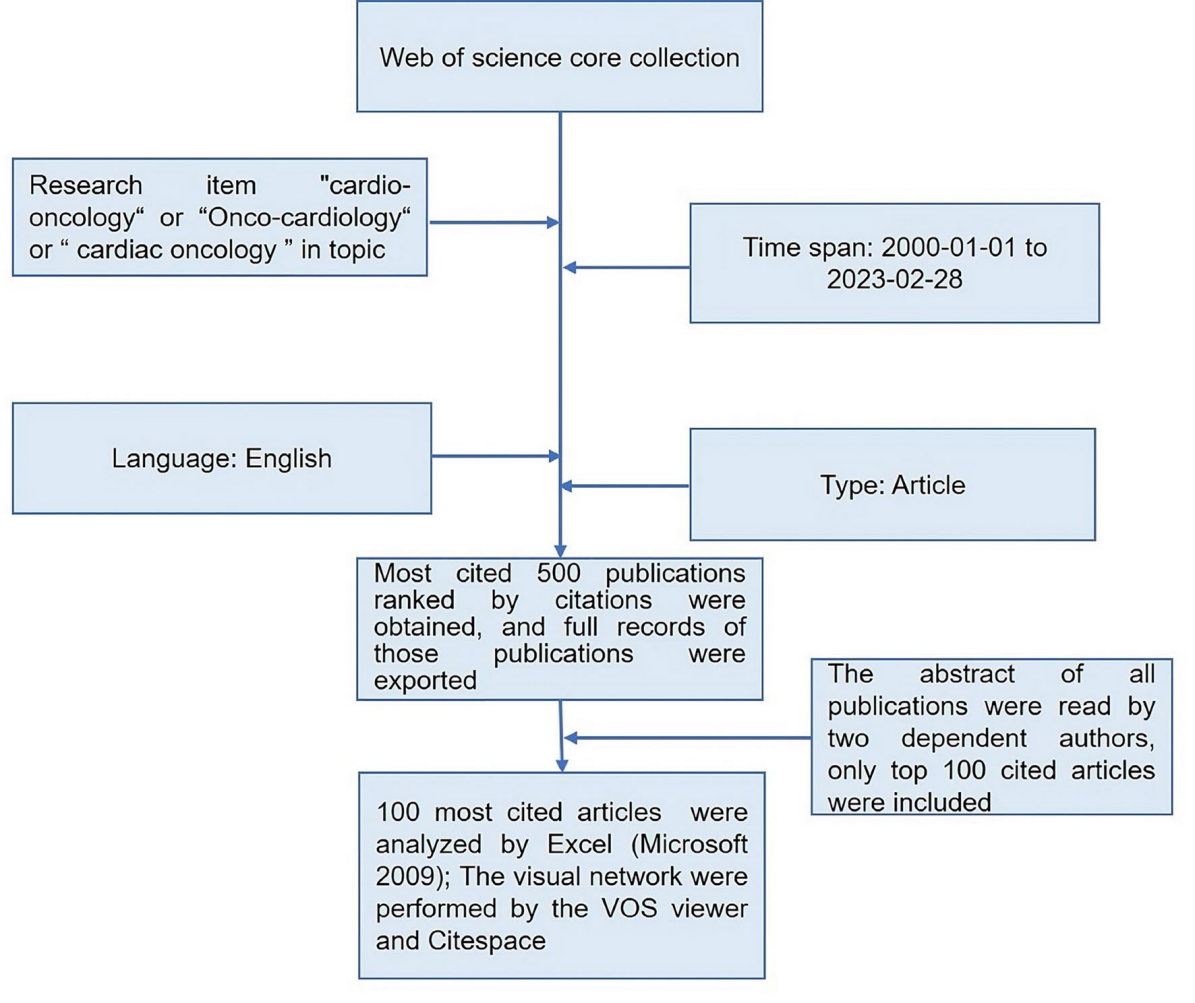
Flow chart of searching and analyzing the top 100 most cited articles

On March 01, 2023, research items TS = *“cardio-oncology or onco-cardiology or cardio-oncology”* were performed by the Web of Science core collection (WoSCC). The selected time frame for this exploration spanned from January 1st, 2000 to February 28th, 2023. Inclusion and Exclusion Criteria: only publications of articles in English were included. This timeframe was chosen to capture developments in the field of cardio-oncology over the past two decades, beginning from the early 2000s when the field began to gain prominence. Full records including all authors, titles, time of publication, journal title, affiliations, keywords, citations, and references of the first 500 records were exported as Excel files. Exported papers were ordered by citations and the abstract was further read by two independent authors to make sure only research papers were included in the final analysis (Flow chart was shown in Fig. 1). Any disputes are decided by the corresponding authors.

### Data analysis

The publications and citations over time, average citation, the distribution of countries or regions, top authors, research institutions, research areas, and journals were calculated in Excel (Microsoft 2009). The number of publications (Np) was used to decide the production of the author, country, and journal. The average published year (APY) was adapted to evaluate the novelty of the keywords. The number of citations without self-citations (Nc) and average citation number (ACN) calculated by total citations/total publications were used to show the influence of the authors, countries, or journals. The distribution of journal and impact factor were got from the journal cited report (2022) which were also adapted to demonstrate the influence of the journal. The main bibliometric indicators include the total amount of publications, the authors, institutions, countries, and citations.

Publications and citations for each author, country, institution, and journal were analyzed by Excel (Microsoft 2009). The visual network for co-citation, authors, countries, and all author keywords was performed by VOSviewer (v.1.6.18, CWTS, Leiden University) based on JAVA. Specific parameter settings were as follows: (a) Co-authorship network: A threshold was set to require each author to have at least 2 publications. (b) Keyword co-occurrence analysis: Keywords must have appeared at least 3 times. Other parameters can be found in the figure legends. CtieSpace (6.1.R3) was employed for detecting clusters of keywords from publications with high citation bursts. CiteSpace parameters were configured as follows: time slicing: 1-year intervals; pruning: pathfinder and pruning of isolated nodes; keyword selection: keywords with high citation bursts were prioritized for cluster detection [22].

## Results

### Overview of T100

**Fig. 2:**
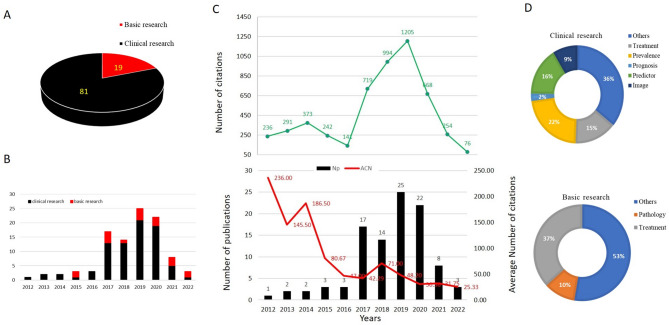
**(A)** the consist of the top 100 most cited articles. **(B)** Time distribution of clinical research articles and basic research articles. **(C)** Total citations of the 100 most cited articles per year (top), and total publications and average citations per year (bottle). **(D)** Main themes of clinical research (top) and basic research (bottle)

T100 comprises 81 clinical research papers and 19 basic research papers published between 2012 and 2022 (Fig. 2A). The majority (78 out of 100) of these publications occurred between 2017 and 2020 (Fig. 2B), highlighting a significant concentration of research output during this period. This surge in publications may suggest increased research activity or interest in the subject matter during these years.

The total citation count for T100 is 5,199, with an annual citation range from 76 to 1,205, and an average of 472.64 citations per year. However, it is important to note the downward trend in citation frequency per year, with average citations decreasing from 236 in 2012 to just 25 in 2022. The exceptions in 2014 and 2018 indicate potential spikes in interest during those years (Fig. 2C).

Among the clinical studies, 22% focus on prognosis, 15% address prevalence, and the remaining 36% cover various other themes. This distribution reflects a broad interest in the outcomes of the condition under study, though prognosis appears to be the most prominent theme within clinical research. For basic research, 10% of papers explore pathology, while 37% focus on treatment strategies. The remaining papers delve into a variety of other research areas. The prominence of treatment-focused research suggests an ongoing exploration of therapeutic options or interventions (Fig. 2D).

### Publication trends across journals

**Fig. 3:**
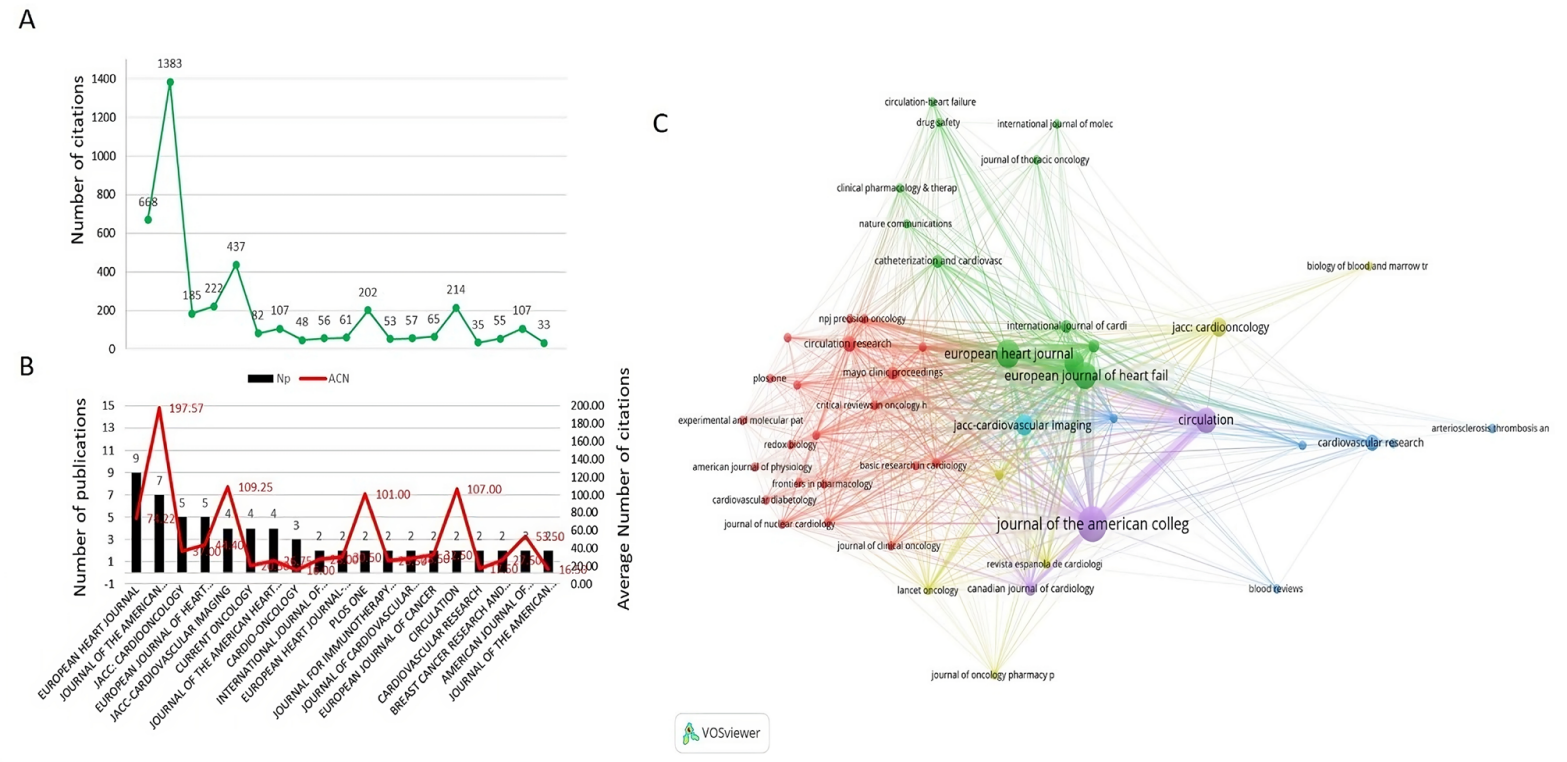
**(A)** Total citations of the journals are more than 2 of T100, and **(B)** Total publications and average citations of the journals are more than 2 of T100 (bottle). **(C)** Visual network map of journals published T100, different color represents different clusters, bigger size of the dot stands for the more publications, lines mean the association between journals

T100 research was published in 56 different journals, with 16 journals accounting for multiple papers. The journals with the most publications include the European Heart Journal and the Journal of the American College of Cardiology, which together contribute significantly to the citation count (over 2,000 citations combined). These journals are prominent in the field, with 13 publications from journals ranked in the Q1 tier (Tables 1, 2; Fig. 3A and B).

**Table 1:** The information of T100 included in current study

Rank	Authors		Journal	Citations	Year	DOI	PMID
1	Mahmood, SS et al.	myocarditis in patients treated with immune checkpoint inhibitors	Journal of the American College of Cardiology	467	2018	10.1016/j.jacc.2018.02.037	29,567,210
2	Ky, B et al.	early increases in multiple biomarkers predict subsequent cardiotoxicity in patients with breast cancer treated with doxorubicin, taxanes, and trastuzumab	Journal of the American College of Cardiology	359	2014	10.1016/j.jacc.2013.10.061	24,291,281
3	Sturgeon, KM et al.	a population-based study of cardiovascular diseasemortality risk in us cancer patients	European Heart Journal	290	2019	10.1093/eurheartj/ehz766	31,761,945
4	Drafts, BC et al.	low to moderate dose anthracycline-based chemotherapy is associated with early noninvasive imaging evidence of subclinical cardiovascular disease	JACC-Cardiovascular Imaging	262	2013	10.1016/j.jcmg.2012.11.017	23,643,285
5	Chen, J et al.	incidence of heart failure or cardiomyopathy after adjuvant trastuzumab therapy for breast cancer	Journal of the American College of Cardiology	236	2012	10.1016/j.jacc.2012.07.068	23,158,536
6	Pituskin, E et al.	multidisciplinary approach to novel therapies in cardio-oncology research (manticore 101-breast): a randomized trial for the prevention of trastuzumab-associated cardiotoxicity	Journal of Clinical Oncology	217	2017	10.1200/JCO.2016.68.7830	27,893,331
7	Focaccetti, C et al.	effects of 5-fluorouracil on morphology, cell cycle, proliferation, apoptosis, autophagy and ros production in endothelial cells and cardiomyocytes	Plos One	176	2015	10.1371/journal.pone.0115686	25,671,635
8	Bonaca, MP et al.	myocarditis in the setting of cancer therapeutics proposed case definitions for emerging clinical syndromes in cardio-oncology	Circulation	176	2019	10.1161/CIRCULATIONAHA.118.034497	31,390,169
9	Galan-Arriola, C et al.	serial magnetic resonance imaging to identify early stages of anthracycline-induced cardiotoxicity	Journal of the American College of Cardiology	122	2019	10.1016/j.jacc.2018.11.046	30,784,671
10	Salem, JE et al.	cardiovascular toxicities associated with ibrutinib	Journal of the American College of Cardiology	111	2019	10.1016/j.jacc.2019.07.056	31,558,250
11	Pareek, N et al.	activity and outcomes of a cardio-oncology service in the United Kingdom-a five-year experience	European Journal of Heart Failure	83	2018	10.1002/ejhf.1292	30,191,649
12	Lopez-Sendon, J et al.	classification, prevalence, and outcomes of anticancer therapy-induced cardiotoxicity: the cardiotox registry	European Heart Journal	76	2020	10.1093/eurheartj/ehaa006	32,016,393
13	Akolkar, G et al.	vitamin c mitigates oxidative/nitrosative stress and inflammation in doxorubicin-induced cardiomyopathy	American Journal of Physiology-Heart and Circulatory Physiology	73	2017	10.1152/ajpheart.00253.2017	28,710,069
14	Quagliariello, V et al.	the sglt-2 inhibitor empagliflozin improves myocardial strain, reduces cardiac fibrosis and pro-inflammatory cytokines in non-diabetic mice treated with doxorubicin	Cardiovascular Diabetology	71	2021	10.1186/s12933-021-01346-y	34,301,253
15	Weberpals, J et al.	long-term heart-specificmortality among 347 476 breast cancer patients treated with radiotherapy or chemotherapy: a registry-based cohort study	European Heart Journal	63	2018	10.1093/eurheartj/ehy167	29,635,274
16	Stoltzfus, KC et al.	fatal heart disease among cancer patients	Nature Communications	61	2020	10.1038/s41467-020-15639-5	32,332,714
17	Henry, ML et al.	cardiotoxicity and cardiac monitoring among chemotherapy-treated breast cancer patients	JACC-Cardiovascular Imaging	63	2018	10.1016/j.jcmg.2018.06.005	30,092,967
18	Finkelman, BS et al.	arginine-nitric oxide metabolites and cardiac dysfunction in patients with breast cancer	Journal of the American College of Cardiology	64	2017	10.1016/j.jacc.2017.05.019	28,683,962
19	Faber, J et al.	burden of cardiovascular risk factors and cardiovascular disease in childhood cancer survivors: data from the german cvss-study	European Heart Journal	58	2018	10.1093/eurheartj/ehy026	29,534,171
20	Narayan, HK et al.	noninvasive measures of ventricular-arterial coupling and circumferential strain predict cancer therapeutics-related cardiac dysfunction	JaACC-Cardiovascular Imaging	61	2016	10.1016/j.jcmg.2015.11.024	27,085,442
21	Demissei, BG et al.	changes in cardiovascular biomarkers with breast cancer therapy and associations with cardiac dysfunction	Journal of the American Heart Association	58	2020	10.1161/JAHA.119.014708	31,959,034
22	D’Souza, M et al.	the risk of cardiac events in patients receiving immune checkpoint inhibitors a nationwide danish study	European Heart Journal	55	2021	10.1093/eurheartj/ehaa884	33,291,147
23	Lefebvre, B et al.	cardiovascular effects of car t cell therapy a retrospective study	JACC: Cardiooncology	54	2020	10.1016/j.jaccao.2020.04.012	32,776,016
24	Vaduganathan, M et al.	efficacy of neurohormonal therapies in preventing cardiotoxicity in patients with cancer undergoing chemotherapy	JACC: Cardiooncology	55	2019	10.1016/j.jaccao.2019.08.006	33,083,790
25	Cappetta, D et al.	effects of ranolazine in a model of doxorubicin-induced left ventricle diastolic dysfunction	British Journal of Pharmacology	50	2017	10.1111/bph.13791	28,320,043
26	Upshaw, JN et al.	comprehensive assessment of changes in left ventricular diastolic function with contemporary breast cancer therapy	JACC-Cardiovascular Imaging	51	2020	10.1016/j.jcmg.2019.07.018	31,542,526
27	Abdel-Qadir, H et al.	the risk of myocardial infarction with aromatase inhibitors relative to tamoxifen in post-menopausal women with early stage breast cancer	European Journal of Cancer	48	2016	10.1016/j.ejca.2016.08.022	27,693,889
28	Huang, H et al.	accuracy of left ventricular ejection fraction by contemporary multiple gated acquisition scanning in patients with cancer: comparison with cardiovascular magnetic resonance	Journal of Cardiovascular Magnetic Resonance	44	2017	10.1186/s12968-017-0348-4	28,335,788
29	Abdel-Qadir, H et al.	development and validation of amultivariable prediction model for major adverse cardiovascular events after early stage breast cancer: a population-based cohort study	European Heart Journal	43	2019	10.1093/eurheartj/ehz460	31,318,428
30	Tabata, N et al.	outcome of current and history of cancer on the risk of cardiovascular events following percutaneous coronary intervention: a kumamoto university malignancy and atherosclerosis (kuma) study	European Heart Journal-Quality of Care and Clinical Outcomes	42	2018	10.1093/ehjqcco/qcx047	29,211,884
31	Michel, L et al.	targeting early stages of cardiotoxicity from anti-pd1 immune checkpoint inhibitor therapy	European Heart Journal	40	2022	10.1093/eurheartj/ehab430	34,389,849
32	Keramida, K et al.	longitudinal changes of right ventricular deformation mechanics during trastuzumab therapy in breast cancer patients	European Journal of Heart Failure	42	2019	10.1002/ejhf.1385	30,811,091
33	Fornaro, A et al.	comparison of long-term outcome in anthracycline-related versus idiopathic dilated cardiomyopathy: a single centre experience	European Journal of Heart Failure	40	2018	10.1002/ejhf.1049	29,148,208
34	Serie, DJ et al.	genome-wide association study of cardiotoxicity in the ncctg n9831 (alliance) adjuvant trastuzumab trial	Pharmacogenetics And Genomics	42	2017	10.1097/FPC.0000000000000302	28,763,429
35	Leong, DP et al.	safety of continuing trastuzumab despite mild cardiotoxicity a phase i trial	JACC: Cardiooncology	39	2019	10.1016/j.jaccao.2019.06.004	34,396,157
36	Laubach, JP et al.	a retrospective analysis of 3954 patients in phase 2/3 trials of bortezomib for the treatment of multiple myeloma: towards providing a benchmark for the cardiac safety profile of proteasome inhibition in multiple myeloma	British Journal of Haematology	40	2017	10.1111/bjh.14708	28,466,536
37	Bockstahler, M et al.	heart-specific immune responses in an animal model of autoimmune-related myocarditis mitigated by an immunoproteasome inhibitor and genetic ablation	Circulation	38	2020	10.1161/CIRCULATIONAHA.119.043171	32,160,764
38	Quagliariello, V et al.	cardiotoxicity and pro-inflammatory effects of the immune checkpoint inhibitor pembrolizumab associated to trastuzumab	International Journal of Cardiology	36	2019	10.1016/j.ijcard.2019.05.028	31,160,077
39	Martin-Garcia, A et al.	effectiveness of sacubitril-valsartan in cancer patients with heart failure	Esc Heart Failure	34	2020	10.1002/ehf2.12627	32,022,485
40	Quagliariello, V et al.	low doses of bisphenol a have pro-inflammatory and pro-oxidant effects, stimulate lipid peroxidation and increase the cardiotoxicity of doxorubicin in cardiomyoblasts	Environmental Toxicology and Pharmacology	35	2019	10.1016/j.etap.2019.03.006	30,903,913
41	Bordun, KA et al.	the utility of cardiac biomarkers and echocardiography for the early detection of bevacizumab- and sunitinib-mediated cardiotoxicity	American Journal of Physiology-Heart and Circulatory Physiology	34	2015	10.1152/ajpheart.00172.2015	26,092,985
42	Chan, O et al.	side-effects profile and outcomes of ponatinib in the treatment of chronic myeloid leukemia	Blood Advances	32	2020	10.1182/bloodadvances.2019000268	32,045,474
43	Banke, A et al.	long-term effect of epirubicin on incidence of heart failure in women with breast cancer: insight from a randomized clinical trial	European Journal of Heart Failure	35	2018	10.1002/ejhf.1168	29,493,047
44	Beer, LA et al.	baseline immunoglobulin e levels as a marker of doxorubicin- and trastuzumab-associated cardiac dysfunction	Circulation Research	32	2016	10.1161/CIRCRESAHA.116.309004	27,582,370
45	Feliz-Mosquea, YR et al.	combination of anthracyclines and anti-cd47 therapy inhibit invasive breast cancer growth while preventing cardiac toxicity by regulation of autophagy	Breast Cancer Research and Treatment	33	2018	10.1007/s10549-018-4884-x	30,056,566
46	Akolkar, G et al.	the role of renin angiotensin system antagonists in the prevention of doxorubicin and trastuzumab induced cardiotoxicity	Cardiovascular Ultrasound	32	2015	10.1186/s12947-015-0011-x	25,889,218
47	Geisberg, CA et al.	circulating neuregulin during the transition from stage a to stage b/c heart failure in a breast cancer cohort	Journal of Cardiac Failure	29	2013	10.1016/j.cardfail.2012.11.006	23,273,589
48	Finke, D et al.	early detection of checkpoint inhibitor-associated myocarditis using ga-68-fapi pet/ct	Frontiers In Cardiovascular Medicine	27	2021	10.3389/fcvm.2021.614997	33,718,446
49	Cautela, J et al.	intensified immunosuppressive therapy in patients with immune checkpoint inhibitor-induced myocarditis	Journal For Immunotherapy of Cancer	27	2020	10.1136/jitc-2020-001887	33,298,621
50	Rushton, M et al.	trastuzumab-induced cardiotoxicity: testing a clinical risk score in a real-world cardio-oncology population	Current Oncology	25	2017	10.3747/co.24.3349	28,680,277
51	Goldman, A et al.	adverse cardiovascular and pulmonary events associated with chimeric antigen receptor t-cell therapy	Journal of the American College of Cardiology	24	2021	10.1016/j.jacc.2021.08.044	34,711,339
52	Baptiste, F et al.	high incidence of atrial fibrillation in patients treated with ibrutinib	Open Heart	24	2019	10.1136/openhrt-2019-001049	31,168,393
53	Dolladille, C et al.	late cardiac adverse events in patients with cancer treated with immune checkpoint inhibitors	Journal for Immunotherapy of Cancer	26	2020	10.1136/jitc-2019-000261	31,988,143
54	Skubitz, KM et al.	cardiac safety profile of patients receiving high cumulative doses of pegylated-liposomal doxorubicin: use of left ventricular ejection fraction is of unproven value	Cancer Chemotherapy and Pharmacology	24	2017	10.1007/s00280-017-3420-8	28,856,562
55	Wang, L et al.	long-term cardiovascular disease mortality among 160 834 5-year survivors of adolescent and young adult cancer: an american population-based cohort study	European Heart Journal	22	2021	10.1093/eurheartj/ehaa779	33,156,911
56	Zhao, R et al.	early detection and prediction of anthracycline-induced right ventricular cardiotoxicity by 3-dimensional echocardiography	JACC: Cardiooncology	21	2020	10.1016/j.jaccao.2020.01.007	34,396,205
57	Tromp, J et al.	long-term survivors of early breast cancer treated with chemotherapy are characterized by a pro-inflammatory biomarker profile compared to matched controls	European Journal of Heart Failure	22	2020	10.1002/ejhf.1758	32,078,215
58	Veronese, P et al.	effects of anthracycline, cyclophosphamide and taxane chemotherapy on qtc measurements in patients with breast cancer	Plos One	26	2018	10.1371/journal.pone.0196763	29,723,224
59	Sourdon, J et al.	cardiac metabolic deregulation induced by the tyrosine kinase receptor inhibitor sunitinib is rescued by endothelin receptor antagonism	Theranostics	21	2017	10.7150/thno.19551	28,824,714
60	Guha, A et al.	incidence, risk factors, and mortality of atrial fibrillation in breast cancer: a seer-medicare analysis	European Heart Journal	21	2022	10.1093/eurheartj/ehab745	34,791,123
61	Galan-Arriola, C et al.	remote ischaemic preconditioning ameliorates anthracycline-induced cardiotoxicity and preserves mitochondrial integrity	Cardiovascular Research	20	2021	10.1093/cvr/cvaa181	32,597,960
62	Guha, A et al. Addison, D et al.	cardiovascular events associated with chimeric antigen receptor t cell therapy: cross-sectional fda adverse events reporting system analysis	Biology of Blood and Marrow Transplantation	20	2020	10.1016/j.bbmt.2020.08.036	32,966,880
63	Hussain, Y et al.	cardiac outcomes of trastuzumab therapy in patients with her2-positive breast cancer and reduced left ventricular ejection fraction	Breast Cancer Research and Treatment	22	2019	10.1007/s10549-019-05139-6	30,721,443
64	Laufer-Perl, M et al.	usefulness of global longitudinal strain for early identification of subclinical left ventricular dysfunction in patients with active cancer	American Journal of Cardiology	24	2018	10.1016/j.amjcard.2018.08.019	30,217,373
65	Catino, AB et al.	longitudinal assessment of vascular function with sunitinib in patients with metastatic renal cell carcinoma	Circulation-Heart Failure	21	2018	10.1161/CIRCHEARTFAILURE.117.004408	29,664,405
66	Calabrese, V et al.	early diastolic dysfunction after cancer chemotherapy: primary endpoint results of a multicenter cardio-oncology study	Chemotherapy	21	2018	10.1159/000486761	29,428,939
67	Law, W et al.	the framingham risk score underestimates the risk of cardiovascular events in the her2-positive breast cancer population	Current Oncology	20	2017	10.3747/co.24.3684	29,089,804
68	Jovenaux, L et al.	practices in management of cancer treatment-related cardiovascular toxicity: a cardio-oncology survey	International Journal of Cardiology	20	2017	10.1016/j.ijcard.2017.02.154	28,365,180
69	Gregorietti, V et al.	use of sacubitril/valsartan in patients with cardio toxicity and heart failure due to chemotherapy	Cardio-Oncology	20	2020	10.1186/s40959-020-00078-4	33,292,750
70	Groarke, JD et al.	association of post-diagnosis cardiorespiratory fitness with cause-specific mortality in cancer	European Heart Journal-Quality of Care and Clinical Outcomes	19	2020	10.1093/ehjqcco/qcaa015	32,167,560
71	Singh, JP et al.	association of cardiac resynchronization therapy with change in left ventricular ejection fraction in patients with chemotherapy-induced cardiomyopathy	Jama-Journal of The American Medical Association	21	2019	10.1001/jama.2019.16658	31,714,987
72	Kosalka, P et al.	effect of obesity, dyslipidemia, and diabetes on trastuzumab-related cardiotoxicity in breast cancer	Current Oncology	21	2019	10.3747/co.26.4823	31,285,674
73	Velders, MA et al.	temporal trends in the prevalence of cancer and its impact on outcome in patients with first myocardial infarction: a nationwide study	Journal of the American Heart Association	18	2020	10.1161/JAHA.119.014383	32,067,596
74	Sheppard, CE et al.	the use of sacubitril/valsartan in anthracycline-induced cardiomyopathy: a mini case series	Journal of Oncology Pharmacy Practice	19	2019	10.1177/1078155218783238	29,945,530
75	Khouri, MG et al.	echocardiography core laboratory reproducibility of cardiac safety assessments in cardio-oncology	Journal of the American Society of Echocardiography	18	2018	10.1016/j.echo.2017.11.018	29,395,626
76	Gronich, N et al.	tyrosine kinase-targeting drugs-associated heart failure	British Journal of Cancer	18	2017	10.1038/bjc.2017.88	28,399,109
77	Gong, JY et al.	immune checkpoint inhibitors for cancer and venous thromboembolic events	European Journal of Cancer	17	2021	10.1016/j.ejca.2021.09.010	34,662,835
78	Quagliariello, V et al.	polydatin reduces cardiotoxicity and enhances the anticancer effects of sunitinib by decreasing pro-oxidative stress, pro-inflammatory cytokines, and nlrp3 inflammasome expression	Frontiers In Oncology	18	2021	10.3389/fonc.2021.680758	34,178,667
79	Laufer-Perl, M et al.	the association of reduced global longitudinal strain with cancer therapy-related cardiac dysfunction among patients receiving cancer therapy	Clinical Research in Cardiology	17	2020	10.1007/s00392-019-01508-9	31,214,777
80	Gulati, G et al.	neurohormonal blockade and circulating cardiovascular biomarkers during anthracycline therapy in breast cancer patients: results from the prada (prevention of cardiac dysfunction during adjuvant breast cancer therapy) study	Journal of the American Heart Association	18	2017	10.1161/JAHA.117.006513	29,118,031
81	Fradley, MG et al.	patterns of anticoagulation use in patients with cancer with atrial fibrillation and/or atrial flutter	JACC: Cardio oncology	16	2020	10.1016/j.jaccao.2020.09.008	34,396,290
82	Novo, G et al.	cardiovascular toxicity in cancer patients treated with tyrosine kinase inhibitors: a real-world single-center experience	Oncology	16	2020	10.1159/000505486	32,348,984
83	Simonetti, V et al.	ozone exerts cytoprotective and anti-inflammatory effects in cardiomyocytes and skin fibroblasts after incubation with doxorubicin	Evidence-Based Complementary and Alternative Medicine	17	2019	10.1155/2019/2169103	31,827,546
84	Upshaw, JN et al.	personalized decision making in early stage breast cancer: applying clinical prediction models for anthracycline cardiotoxicity and breast cancer mortality demonstrates substantial heterogeneity of benefit-harm trade-off	Clinical Breast Cancer	16	2019	10.1016/j.clbc.2019.04.012	31,175,052
85	Kappel, C et al.	clinical experience of patients referred to a multidisciplinary cardio-oncology clinic: an observational cohort study	Current Oncology	16	2019	10.3747/co.26.4509	31,285,675
86	Quagliariello, V et al.	nano-encapsulation of coenzyme q10 in secondary and tertiary nano-emulsions for enhanced cardioprotection and hepatoprotection in human cardiomyocytes and hepatocytes during exposure to anthracyclines and trastuzumab	International Journal of Nanomedicine	15	2020	10.2147/IJN.S245170	32,764,923
87	Peng, J et al.	an international survey of healthcare providers’ knowledge of cardiac complications of cancer treatments	Cardio-Oncology	15	2019	10.1186/s40959-019-0049-2	32,154,018
88	Tabata, N et al.	a retrospective study of arterial stiffness and subsequent clinical outcomes in cancer patients undergoing percutaneous coronary intervention	Journal of Hypertension	15	2019	10.1097/HJH.0000000000001949	30,817,457
89	Nhola, LF et al.	echocardiographic assessment for the detection of cardiotoxicity due to vascular endothelial growth factor inhibitor therapy in metastatic renal cell and colorectal cancers	Journal of the American Society of Echocardiography	15	2019	10.1016/j.echo.2018.09.019	30,459,123
90	Ganatra, S et al.	re-evaluating the safety of drug-eluting stents in cancer patients	JACC-Cardiovascular Interventions	15	2017	10.1016/j.jcin.2017.06.068	29,169,502
91	Galan-Arriola, C et al.	coronary microcirculation damage in anthracycline cardiotoxicity	Cardiovascular Research	15	2022	10.1093/cvr/cvab053	33,605,403
92	Miller, JM et al.	heart slice culture system reliably demonstrates clinical drug-related cardiotoxicity	Toxicology And Applied Pharmacology	14	2020	10.1016/j.taap.2020.115213	32,877,659
93	Lentz, R et al.	risk factors for the development of atrial fibrillation on ibrutinib treatment	Leukemia & Lymphoma	15	2019	10.1080/10428194.2018.1533129	30,730,240
94	Sato, A et al.	valvular heart disease as a possible predictor of trastuzumab-induced cardiotoxicity in patients with breast cancer	Molecular and Clinical Oncology	14	2019	10.3892/mco.2018.1764	30,655,975
95	Ibrahim, YF et al.	docetaxel reverses pulmonary vascular remodeling by decreasing autophagy and resolves right ventricular fibrosis	Journal of Pharmacology and Experimental Therapeutics	15	2017	10.1124/jpet.117.239921	28,760,737
96	Lenneman, CG et al.	sympathetic nervous system alterations with her2 + antagonism: an early marker of cardiac dysfunction with breast cancer treatment?	Ecancermedicalscience	14	2014	10.3332/ecancer.2014.446	25,114,718
97	Zhou, YD et al.	machine learning-based risk assessment for cancer therapy-related cardiac dysfunction in 4300 longitudinal oncology patients	Journal of the American Heart Association	13	2020	10.1161/JAHA.120.019628	33,241,727
98	Clark, RA et al.	cardiotoxicity after cancer treatment: a process map of the patient treatment journey	Cardio-Oncology	13	2019	10.1186/s40959-019-0046-5	32,154,020
99	Sanz, AP et al.	current status of anticoagulation in patients with breast cancer and atrial fibrillation	Breast	13	2019	10.1016/j.breast.2019.05.017	31,220,790
100	Chan, AT et al.	prognostic utility of differential tissue characterization of cardiac neoplasm and thrombus via late gadolinium enhancement cardiovascular magnetic resonance among patients with advanced systemic cancer	Journal of Cardiovascular Magnetic Resonance	13	2017	10.1186/s12968-017-0390-2	29,025,425

**Table 2:** Journals published over two papers among the T100

Ranked	Journals	Nc	Np	ACN	JCR	IF(2021)
1	European Heart Journal	668	9	74.22	Q1	35.86
2	Journal of the American College Of Cardiology	1383	7	197.57	Q1	27.21
3	Jacc: Cardiooncology	185	5	37.00	Q1	8.42
4	European Journal of Heart Failure	222	5	44.40	Q1	18.17
5	Jacc-Cardiovascular Imaging	437	4	109.25	Q1	16.05
6	Current Oncology	82	4	20.50	Q2	3.11
7	Journal of the American Heart Association	107	4	26.75	Q1	6.11
8	Cardio-Oncology	48	3	16.00	Q2	3.57
9	International Journal of Cardiology	56	2	28.00	Q2	4.04
10	European Heart Journal-Quality of Care and Clinical Outcomes	61	2	30.50	Q1	7.06
11	Plos One	202	2	101.00	Q2	3.42
12	Journal For Immunotherapy of Cancer	53	2	26.50	Q1	12.49
13	Journal of Cardiovascular Magnetic Resonance	57	2	28.50	Q1	6.90
14	European Journal of Cancer	65	2	32.50	Q1	10.00
15	Circulation	214	2	107.00	Q1	39.92
16	Cardiovascular Research	35	2	17.50	Q1	14.24
17	Breast Cancer Research and Treatment	55	2	27.50	Q2	4.85
18	American Journal of Physiology-Heart and Circulatory Physiology	107	2	53.50	Q2	4.73
19	Journal of the American Society of Echocardiography	33	2	16.50	Q1	7.72

Note: Np: number of publications, Nc: number of citations without self-citations, ACN: average citation number, JCR: journal citation report, IF: impact factor

Interestingly, journals such as Journal of the American College of Cardiology, JACC-Cardiovascular Imaging, Circulation, and PLOS One each have an average citation number (ACN) exceeding 100, making them influential platforms in this research area (Fig. 3C). A visualization of the journal network via VOSviewer shows the interconnectedness of key journals, particularly the European Heart Journal and Journal of the American College of Cardiology (Fig. 3C).

### Global contributions to T100

**Fig. 4:**
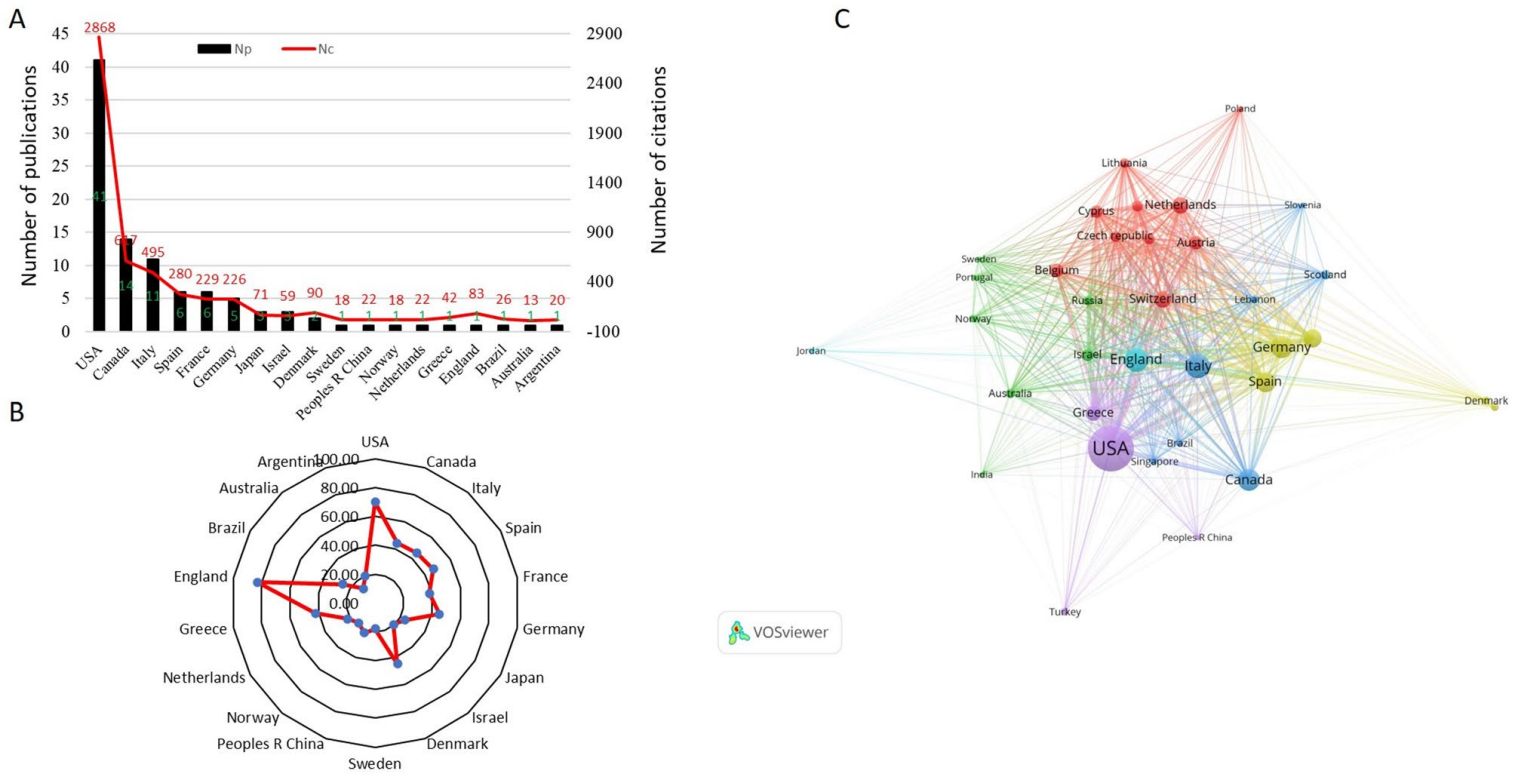
**(A)** total publications and citations of countries published T100, and **(B)** Radar chart of average citations of the countries published T100. **(C)** Visual network map of countries published T100, the wider the circle means the more publications and the thicker the line stands for the closer the cooperation between the countries/regions

T100 was authored by researchers from 18 countries. Notably, the USA leads with 41 papers and a significant citation contribution (2,868 citations), followed by Canada (14 papers, 617 citations) and Italy (11 papers, 495 citations). The USA’s high citation count and extensive collaborations with other nations like England, Germany, and Canada underline its leadership role in this research field (Fig. 4A and B). Furthermore, the USA shows strong international partnerships, as depicted in the co-authorship network (Fig. 4C).

### Author contributions and influence

**Fig. 5:**
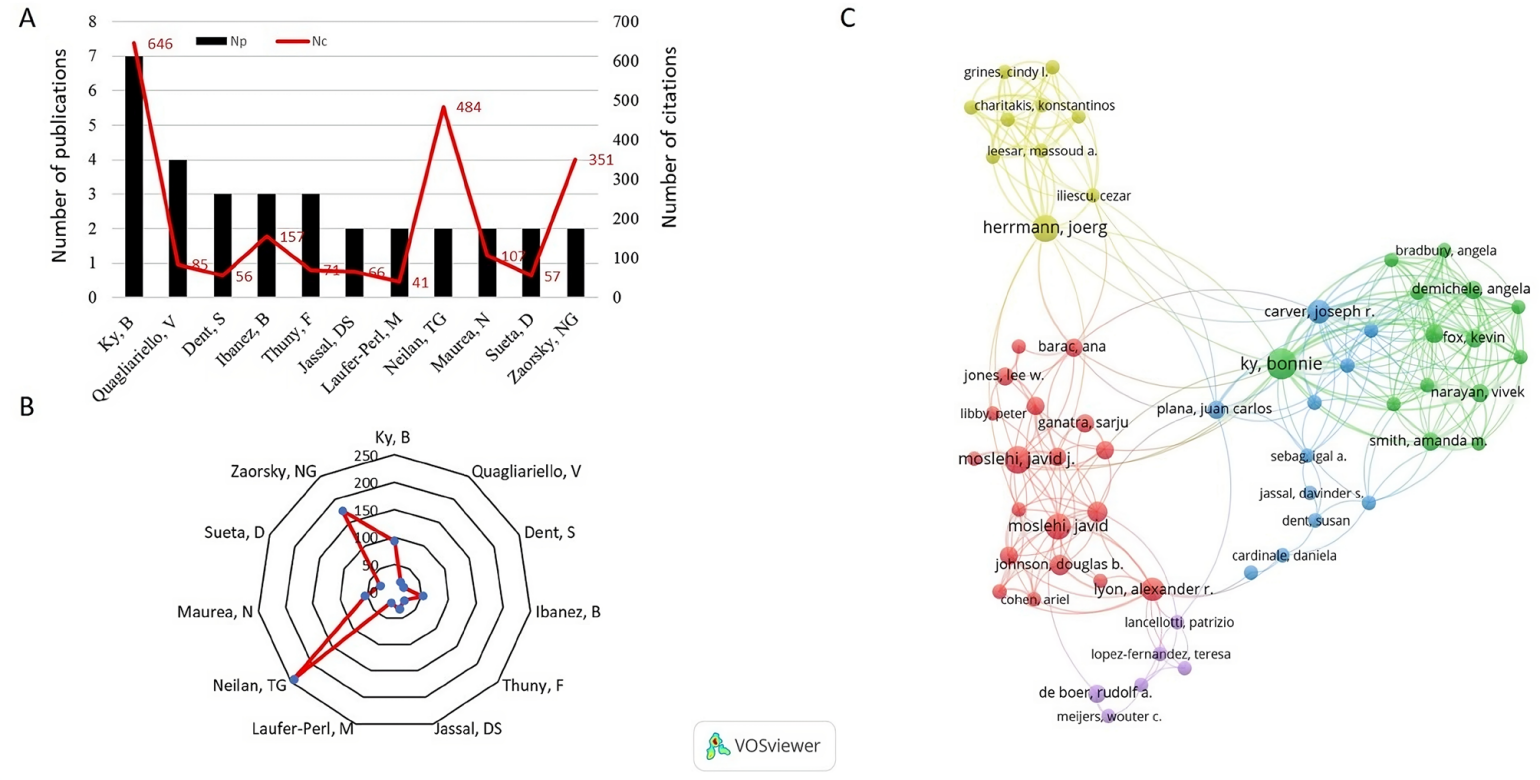
**A.** Total publications and citations of authors, and **B**. Radar chart of average citations of the authors published more than 2 of T100. **C.** Visual network map co-authorship authors with more than 2 of T100, the size of the circle represents the number of publications, and the line stands for the co-authorship between the authors

Over 600 authors contributed to T100, with 11 authors having multiple publications. Among these, Ky, B stands out with 7 papers, 646 citations, and an impressive ACN of 92.30. Notably, the highest ACN is held by Neilan, TG (ACN: 242.00), who has 2 papers with substantial impact. This highlights the substantial contributions of certain key authors to the field (Fig. 5A and B). The co-authorship network map further emphasizes the prominent connections between leading authors, with Ky, B being particularly well-connected (Fig. 5C).

### Institutional contributions

**Fig. 6:**
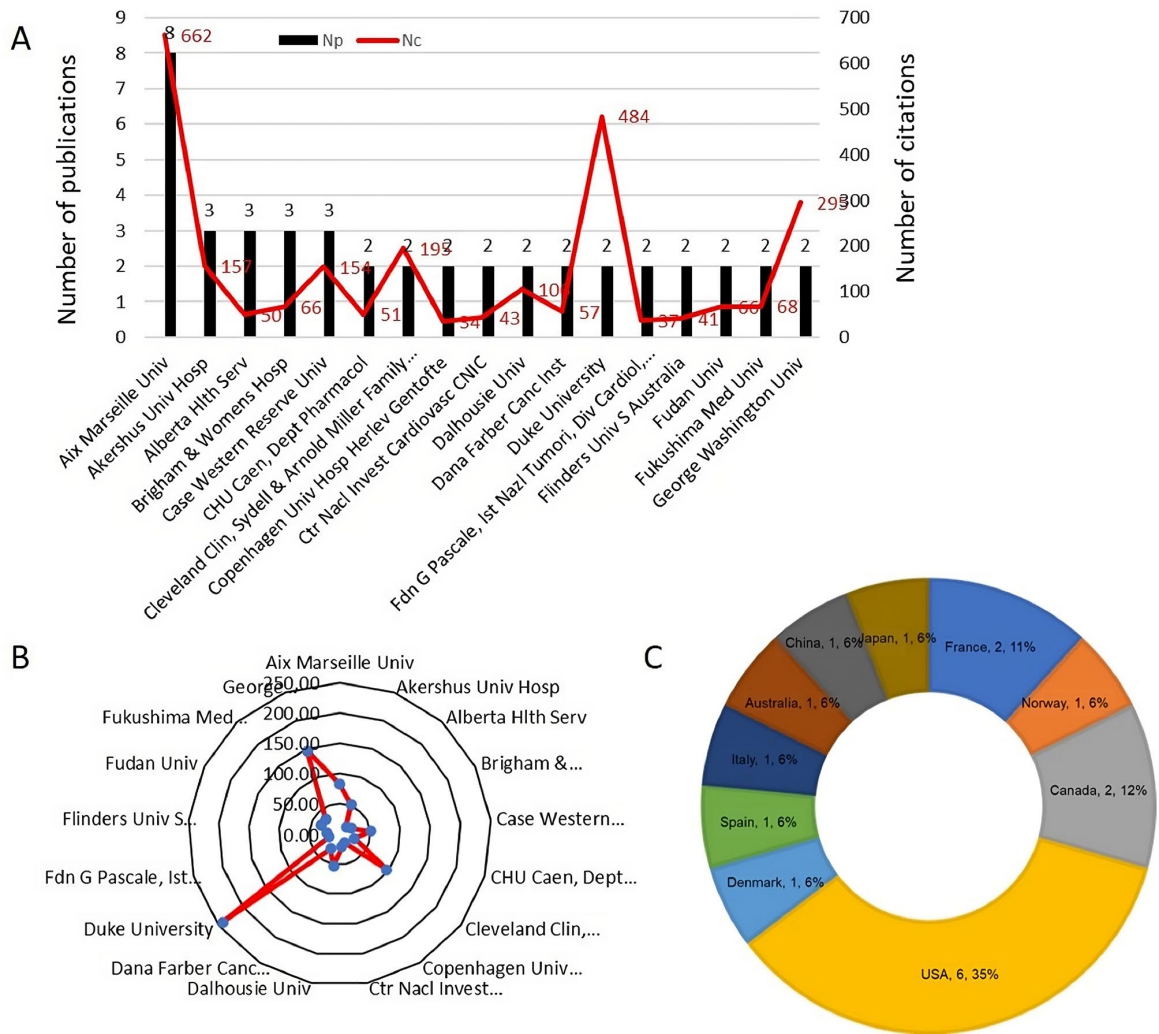
**(A)** Total publications and citations of institutions, and **(B)** Radar chart of average citations of the institutions published more than 2 of T100. **(C)** Country distribution of institutions published more than 2 of T100

T100 includes contributions from 73 institutions, with 17 institutions having published more than two papers. Aix Marseille University, based in the UK, leads in both the number of publications (8 papers) and citations (662 citations). However, in terms of ACN, Duke University holds the top position (ACN: 242.00), followed closely by George Washington University (ACN: 147.50) (Fig. 6A and B). It is notable that 6 of the 17 institutions with multiple publications are based in the USA (Fig. 6C).

### Analysis of keywords from T100 and potential research frontiers

To identify the dominant themes and emerging research trends in heart failure, an in-depth keyword analysis was conducted using VOSviewer and Citespace. This analysis provides a comprehensive view of the evolving focus within the field and highlights potential areas for future research.

**Fig. 7:**
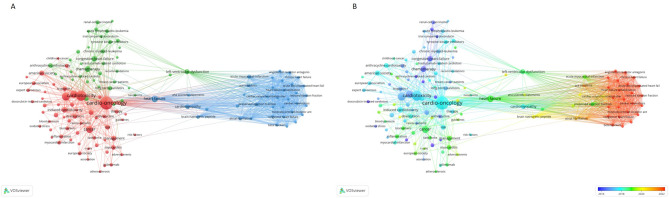
**(A)** Visual network of 134 keywords with more than 3 occurrences formed by VOSviewer. The size of the node stands for the time of occurrence, and lines between dots mean the co-occurrence of keywords. **(B)** Visual network of 134 keywords with more than 3 occurrences over time conducted by VOSviewer. The size of the node stands for the time of occurrence, and lines between dots mean the co-occurrence of keywords. Purple stands for old keywords, and yellow means emerging keywords

A total of 134 keywords, each appearing more than three times, were categorized into three primary clusters (Fig. 7A). Cluster 3 emerged as a focal point for recent advancements, particularly post-2021. It contained keywords related to various types of heart failure, including acute decompensated heart failure, heart failure with mildly reduced ejection fraction, and heart failure with reduced ejection fraction. In Fig. 7B, the colours of all keywords were separated according to the APY. The color of the circle corresponds to the timeline at the lower-right corner of the picture. Additionally, treatment strategies such as beta blockers, mineralocorticoid receptor antagonists, and SGLT2 inhibitors, along with risk factors like valvular heart disease, mitral regurgitation, and cardiomyopathy in pregnancy, demonstrated high APY(around 2022). These trends signify the growing importance of these topics as emerging areas of research in the years following 2021 (Fig. 7B).

**Fig. 8:**
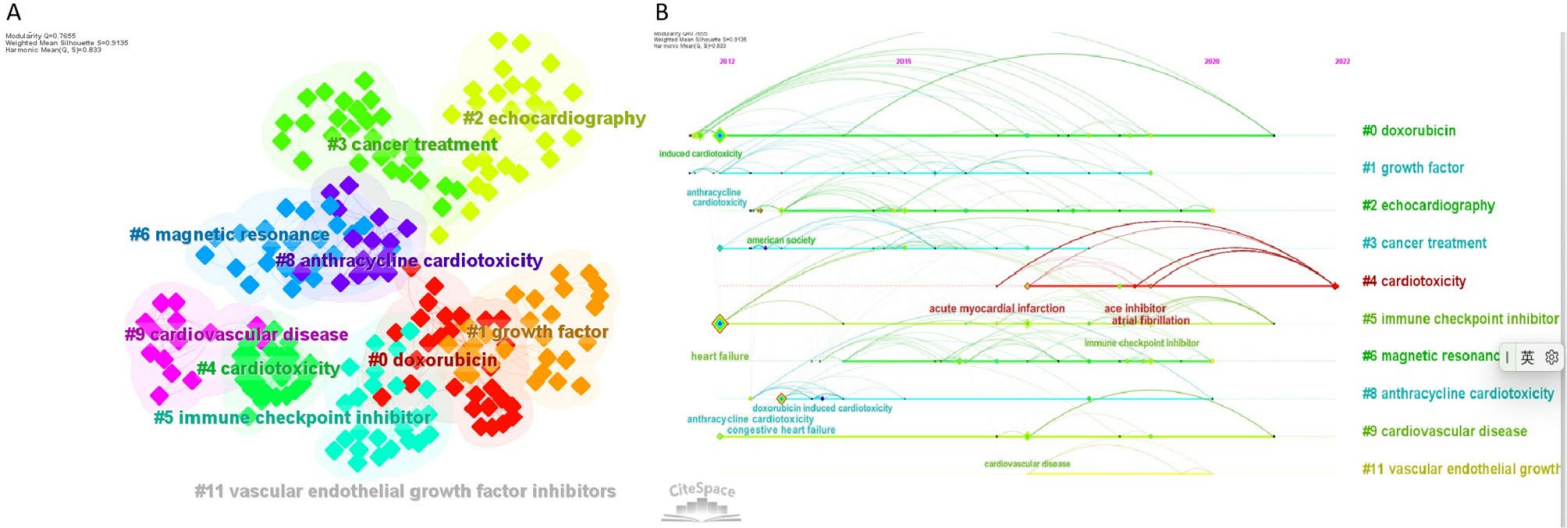
**(A)** Visual analysis of the clusters decided by the keywords of T100 performed by CiteSpace. **(B)** Timeline viewer of keywords from the clusters of keywords from T100 by CiteSpace. Note: In Panel A, nodes in the same color group belong to a thematic cluster, showing groups of closely related keywords. In Panel B, nodes represent keywords, and their size indicates the frequency of occurrence. Links represent co-occurrence or thematic connections over time

Further analysis with Citespace identified 11 keyword clusters, providing a timeline of research evolution across different domains. For example, Cluster #0, centered around dexorubicin, indicates early emphasis on chemotherapy-related cardiac concerns. Cluster #1, focusing on growth factors, and Cluster #2, centered around echocardiography, highlight the long-standing interest in therapeutic interventions and diagnostic tools, respectively (Fig. 8A).

In Cluster #4 (“cardiotoxicity”), the keyword acute myocardial infarction initially dominated, but later was replaced by atrial fibrillation, reflecting a shift in research focus toward long-term cardiac effects of treatment and disease (Fig. 8B). Additionally, in Cluster #5 (“immune checkpoint inhibitors”), heart failure was a central keyword around 2012, but more recent research (post-2020) increasingly features immune checkpoint inhibitors, underscoring a significant shift toward immunotherapy’s role in heart failure management.

To pinpoint the most significant shifts in research interest, we analyzed the top 15 keywords based on their burst intensity and the years in which they peaked. Early T100 focal points included heart failure, chronic myeloid leukemia, breast cancer patient, and anthracycline-related cardiotoxicity. These themes were foundational in the field but have since evolved as new areas gained prominence.

**Fig. 9:**
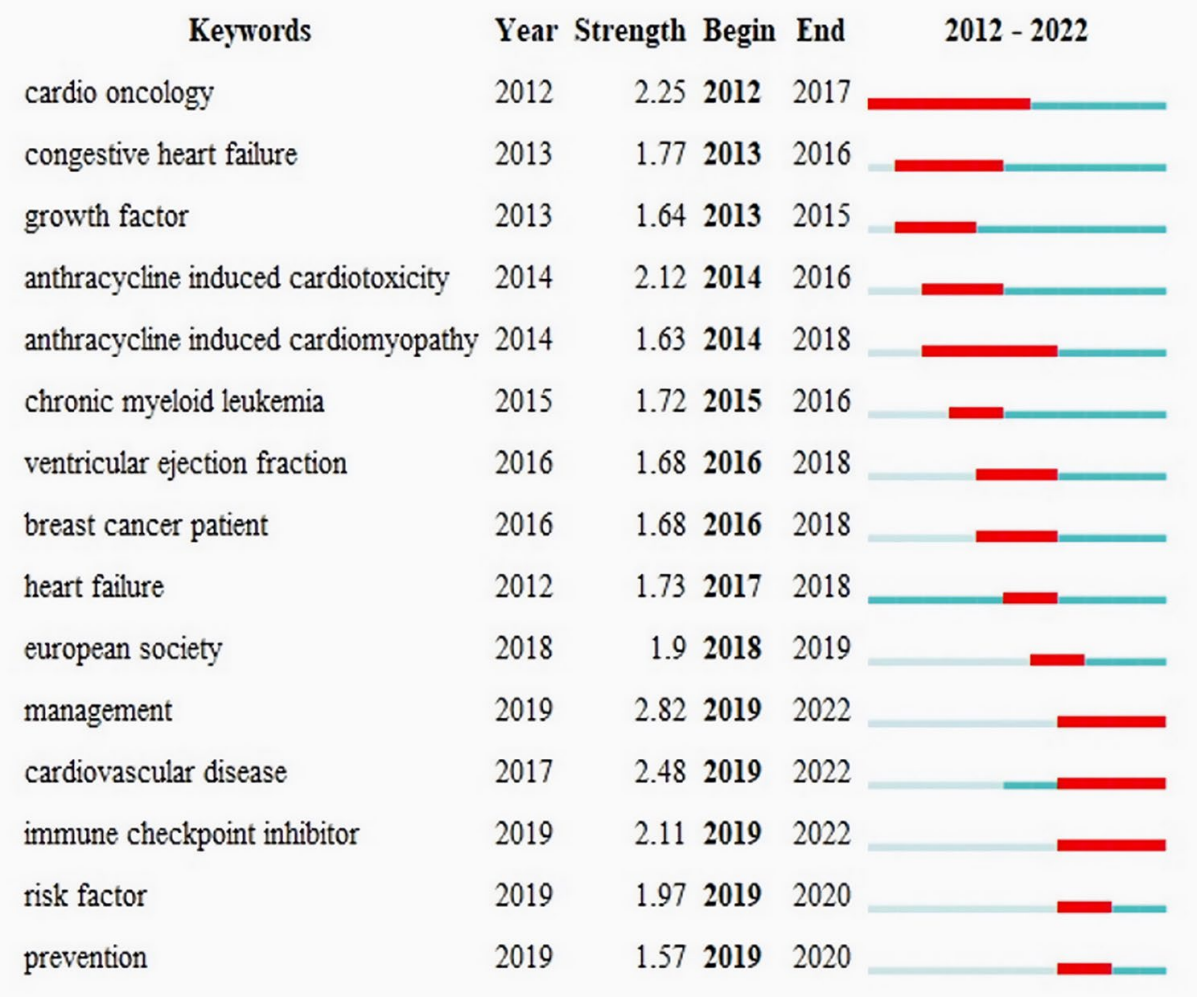
Top 15 keywords with the strongest citation bursts formed by Citespace. Note: Burst intensity in CiteSpace quantifies the speed and magnitude of a node’s (e.g., keyword) rising occurrence over time. High burst marks sudden, sustained research foci (e.g., paradigm shifts), while low burst reflects modest growth, such as nascent trends or niche topics

Recent high-intensity topics include risk factors (strength: 1.97, burst years: 2019–2020), prevention (strength: 1.57, burst years: 2019–2020), cardiovascular diseases (strength: 2.48, burst years: 2019–2022), management (strength: 2.82, burst years: 2019–2022), and immune checkpoint inhibitors (strength: 2.11, burst years: 2019–2022) (Fig. 9). These keywords highlight the shift toward understanding the broader implications of heart failure, emphasizing preventive strategies, the management of comorbidities, and the integration of emerging therapies like immune checkpoint inhibitors.

## Discussion

The present study constitutes the inaugural bibliometric analysis of T100 articles within the realm of cardio-oncology. The primary findings can be distilled into the following key points: (1) Research endeavors in this domain are predominantly centered on clinical investigations. (2) The United States occupies a definitive role in shaping the trajectory of cardio-oncology research. (3) Exploration of underlying mechanisms, the potential of immune checkpoint inhibitors, along with the comprehensive management of cardiovascular diseases such as heart failure and atrial fibrillation, encompassing prevention and treatment modalities, emerge as prominent hotspots within this field.

Research papers are a cornerstone of academic discourse, providing comprehensive analyses, evaluations, and interpretations grounded in empirical evidence. These contributions significantly advance knowledge within specialized fields, offering novel insights and discoveries. By focusing on the T100 research papers, this study highlights the key issues currently dominating the field of cardio-oncology.

It is not surprising that over 80% of the T100 papers were clinical studies. Given that cardio-oncology primarily addresses the cardiovascular side effects of anti-cancer therapies, this area poses significant challenges for clinical practice. Numerous studies have explored epidemiology, risk factors, pretreatment, and treatment in cardio-oncology, forming the foundation of our understanding of the field. However, despite the advancements in clinical research, the underlying mechanisms of cardio-oncology remain largely unexplored. A key area for future research lies in better understanding the relationship between basic research and clinical studies. Basic studies provide the necessary foundation for precise, targeted treatments for CTR-CVT [23].

Interestingly, the European Heart Journal published the most T100 papers, reflecting its strong alignment with the research themes of cardio-oncology. The journal’s high impact factor likely attracts high-quality studies, amplifying its influence within the field. The fact that all T100 papers in this journal were published between 2018 and 2022 emphasizes the journal’s growing prominence [24, 25]. In comparison, The Journal of the American College of Cardiology, ranked second in terms of T100 publications, spans a broader time range (2012–2021), underscoring its historical significance in the field.This aligns with findings from Hernandez et al., who demonstrated that longstanding journals maintain a consistent influence on research trends in cardio-oncology [26, 27]. In addition, studies on predictors [9], and management [28] in cardio-oncology were published in the Journal of the American College of Cardiology, which reflected the wide coverage of this journal in this area.

Nearly half of the T100 papers were authored by U.S.-based researchers, and the U.S. led in both total and average citations, confirming its global leadership in cardio-oncology. The most prolific author among the T100, Ky, B, along with 33 out of 79 authors, was based in the U.S. U.S. institutions, in collaboration with other countries like Canada [29], and Germany [10], played a key role in advancing research.

The rapid progress in cardio-oncology is a result of several factors, including the increasing recognition of cardiotoxicity as a major side effect of cancer therapies [6]. Early studies focused on identifying heart dysfunction in cancer patients and understanding the mechanisms behind these effects [26]. These foundational studies developed a method to precisely assess the heart dysfunction [30], and paved the way for later investigations into risk factors and the specific mechanisms of CTR-CVT [31]. These studies served as a foundation for subsequent research that focused on finding associated risk factors [32] and summarizing the CTR-CVT [33]. Age, gender, smoking, sedentary behavior, and metabolic syndrome are two common risk factors that have been shown to raise the risk of tumors and cardiovascular diseases [34]. Furthermore, growing evidence suggests a bidirectional relationship between cardiovascular disease and cancer, with conditions like heart failure and hypertension promoting tumor growth, and tumors influencing cardiovascular disease [35].

Given the high prevalence of CTR-CVT due to the rising incidence of cancer, it is crucial to develop systemic management strategies that balance cancer treatment with the prevention and management of cardiac side effects. The variability in CTR-CVT across cancer types and treatments underscores the need for personalized management approaches [3]. There is an urgent need for more clinical studies focusing on cardiovascular comorbidities such as heart failure (both reduced and preserved ejection fraction) [36], and arrhythmia [37]. However, studies addressing these issues remain limited.

Since the first antibody blocking an immune checkpoint was authorized, paramount achievements have been achieved in the last decades [38]. The application of ICIs has yielded promising results in the treatment of diverse cancers [39]. The generation of ICIs has increased rapidly, and the using of ICIs has highly increased. Meanwhile, side effects from ICIs therapy are gradually manifesting [40]. While this study emphasizes the importance of ICIs in cardio-oncology, it is crucial to recognize potential alternative perspectives. The rapid growth in the use of ICIs may not only reflect scientific advancements but also be influenced by substantial industry funding, which often drives research priorities.

Furthermore, although myocarditis is a known side effect of ICI therapy, the precise mechanisms behind ICI-induced myocarditis and other cardiovascular complications remain poorly understood [41]. Several potential mechanisms of CVDs following ICIs were reported, but the exact mechanism involved in the development of myocarditis, pericardial disease, arrhythmia, and myocardial infarction were poorly understood [42]. Additionally, there is a pressing need for more rigorous research into the risk factors for CTR-CVT associated with ICI therapy [39]. Long-term follow-up studies and more comprehensive datasets are necessary to identify consistent biomarkers and therapeutic targets for managing these side effects. The application of ICIs will continue to grow, and related studies will be more interesting since the indications for ICIs are rapidly expanding [43].

### Limitation

The current study is subject to several limitations. Firstly, it solely encompasses research articles sourced from WoSSC, potentially leading to variations when compared to data from other databases. Secondly, the evolving nature of paper citations means that the composition of T100 may vary across different timeframes. Thirdly, while author keywords were considered in this analysis, exploring additional keyword categories such as keywords plus could yield divergent outcomes.

## Conclusion

Cardio-oncology is an emerging interdisciplinary field that requires close collaboration between oncologists and cardiologists. While clinical studies have been at the forefront, there is a pressing need to expand basic research to better understand the mechanisms linking cancer therapies and cardiovascular diseases. This will allow for the development of personalized treatment strategies that improve patient outcomes.

Key journals like the European Heart Journal and the Journal of the American College of Cardiology continue to lead the dissemination of impactful research, shaping the future of the field. The United States remains a leader in cardio-oncology research, reflected in its strong publication and citation output.

While heart failure and atrial fibrillation remain central to research, there is a growing focus on holistic management that integrates prevention and treatment strategies. Additionally, the increasing role of immune checkpoint inhibitors presents a promising area for future investigation, with significant potential to advance care in cardio-oncology.

In summary, expanding basic research and exploring emerging therapies like immune checkpoint inhibitors are crucial for advancing both research and clinical practice in cardio-oncology.

## Data Availability

No datasets were generated or analysed during the current study.
